# Distinct Functional Requirements for Podocalyxin in Immature and Mature Podocytes Reveal Mechanisms of Human Kidney Disease

**DOI:** 10.1038/s41598-020-64907-3

**Published:** 2020-06-10

**Authors:** Ido Refaeli, Michael R. Hughes, Alvin Ka-Wai Wong, Mei Lin Z. Bissonnette, Calvin D. Roskelley, A. Wayne Vogl, Sean J. Barbour, Benjamin S. Freedman, Kelly M. McNagny

**Affiliations:** 1 The Biomedical Research Centre, University of British Columbia, Vancouver, BC Canada; 2 Department of Medical Genetics, University of British Columbia, Vancouver, BC Canada; 3 School of Biomedical Engineering, University of British Columbia, Vancouver, BC Canada; 4 Department of Pathology and Laboratory Medicine, University of British Columbia, Vancouver, BC Canada; 5 Life Sciences Institute, Department of Cellular and Physiological Sciences, University of British Columbia, Vancouver, BC Canada; 6 Division of Nephrology, Department of Medicine, University of British Columbia, Vancouver, BC Canada; 7 Division of Nephrology, Department of Medicine, University of Washington School of Medicine, Seattle, WA USA; 8 Kidney Research Institute, University of Washington School of Medicine, Seattle, WA USA; 9 Institute for Stem Cell and Regenerative Medicine, University of Washington School of Medicine, Seattle, WA USA

**Keywords:** Cell biology, Genetics, Physiology, Glomerular diseases

## Abstract

Dominant and recessive mutations in podocalyxin (*PODXL*) are associated with human kidney disease. Interestingly, some *PODXL* mutations manifest as anuria while others are associated with proteinuric kidney disease. *PODXL* heterozygosity is associated with adult-onset kidney disease and podocalyxin shedding into the urine is a common biomarker of a variety nephrotic syndromes. It is unknown, however, how various lesions in *PODXL* contribute to these disparate disease pathologies. Here we generated two mouse stains: one that deletes *Podxl* in developmentally mature podocytes (*Podxl*^∆Pod^) and a second that is heterozygous for podocalyxin in all tissues (*Podxl*^+/−^). We used histologic and ultrastructural analyses, as well as clinical chemistry assays to evaluate kidney development and function in these strains. In contrast to null knockout mice (*Podxl*^−/−^), which die shortly after birth from anuria and hypertension, *Podxl*^∆Pod^ mice develop an acute congenital nephrotic syndrome characterized by focal segmental glomerulosclerosis (FSGS) and proteinuria. *Podxl*^+/−^ mice, in contrast, have a normal lifespan, and fail to develop kidney disease under normal conditions. Intriguingly, although wild-type C57Bl/6 mice are resistant to puromycin aminonucleoside (PA)-induced nephrosis (PAN), *Podxl*^+/−^ mice are highly sensitive and PA induces severe proteinuria and collapsing FSGS. In summary, we find that the developmental timepoint at which podocalyxin is ablated (immature vs. mature podocytes) has a profound effect on the urinary phenotype due to its critical roles in both the formation and the maintenance of podocyte ultrastructure. In addition, *Podxl*^∆Pod^ and *Podxl*^+/−^ mice offer powerful new mouse models to evaluate early biomarkers of proteinuric kidney disease and to test novel therapeutics.

## Introduction

Podocalyxin (human: *PODXL*; mouse: *Podxl*) is a single-pass transmembrane sialomucin whose expression in the kidney is restricted to the surface of podocytes and vascular endothelial cells^1^. It is a member of the CD34 family of stem cell sialomucins and is characterized by a negatively charged extracellular mucin domain (owing to the protein’s extensive O- and N-linked glycosylation and sialic acid modifications), and an intracellular domain that contains an ezrin/radixin/moesin (ERM) binding sequence and a C-terminal PDZ domain docking site, each of which facilitates linkage to the actin cytoskeleton^2–8^. In the developing human and mouse kidney, Podxl controls the morphogenesis and differentiation of nascent podocytes through dissolution of tight and adherens junctions and the subsequent formation of microvilli, foot processes and slit diaphragms—the key ultrastructural elements that enable glomerular filtration^9–12^. In mice, homozygous loss of *Podxl* leads to perinatal retention of junctional complexes between immature podocytes, a walling off of the urinary space, renal failure and, ultimately, perinatal death^9^. Compound heterozygous null mutations in human *PODXL* also lead to a congenital nephrotic syndrome and anuria similar to that observed in *Podxl* null mice^13^. Beyond this early developmental role for Podxl in podocyte morphogenesis, whether or not it directly serves additional functions in mature podocytes and is dysfunctional in adult renal disease remains to be determined.

FSGS, a podocyte-driven disease, is the most common diagnosis in patients biopsied for kidney abnormalities, and frequently progresses to end-stage renal disease (ESRD)^14,15^. FSGS can be idiopathic, secondary to a variety of defined insults or hyperfiltration, or a result of genetic mutations in genes that regulate podocyte structure or function^16,17^. Generally, autosomal recessive inheritance patterns with homozygous or compound heterozygous mutations associate with kidney disease in early childhood, while autosomal dominant inheritance patterns exhibit later onset and have greater clinical variability. Recently, three familial studies have linked dominant and recessive mutations in *PODXL* to human renal disease^13,18,19^. In one study, heterozygous nonsense mutations with autosomal dominant inheritance co-segregated with disease in affected members of a family with adult-onset renal insufficiency and proteinuria^19^. In another, autosomal recessive mutations in the gene caused congenital nephrotic syndrome^13^. In summary, there is evidence for *PODXL* lesions driving both early and late onset kidney disease^20^.

It is not immediately apparent why some *Podxl* mutations associate with proteinuric kidney disease, while others cluster with anuria. A survey of the literature indicates that, possibly, patients with heterozygous null mutations develop proteinuria over a longer time-course^19^, while homozygous null mutations present with congenital anuria and rapidly progressing disease^9,13^. Previously, we generated the first *Podxl*-knockout mouse using homologous recombination^9^. In this strain, *Podxl* was deleted in embryonic stem cells prior to implantation, and the resulting anuric phenotype reflected gene deletion prior to podocyte maturation. In the present  study, to better clarify the contribution of *Podxl* mutations to mature podocyte function, we have conditionally deleted *Podxl* from terminally differentiated, capillary loop stage (CLS) mouse podocytes. We find that these (*Podxl*^∆Pod^) mice survive to approximately 3–7 weeks of age and present with severe proteinuria, FSGS, effacement of podocyte foot processes and a failure to target apical and basal proteins to the appropriate cellular domain. In contrast, *Podxl*^+/−^ mice have no obvious renal phenotype at steady state, but are highly susceptible to chemically-induced nephrosis and phenocopy the pathology observed in patients with adult-onset collapsing FSGS. These later findings provide support of a two-hit mechanism (one genetic and one environmental) in the onset of adult FSGS, and highlight the *Podxl*^+/−^ mouse strain as a novel model for the development of biomarkers of FSGS and as a screening tool for the preclinical evaluation of therapeutics for proteinuric kidney disease.

## Results

### Loss of podocalyxin from capillary loop-stage podocytes results in congenital nephrotic syndrome

**Figure 1:**
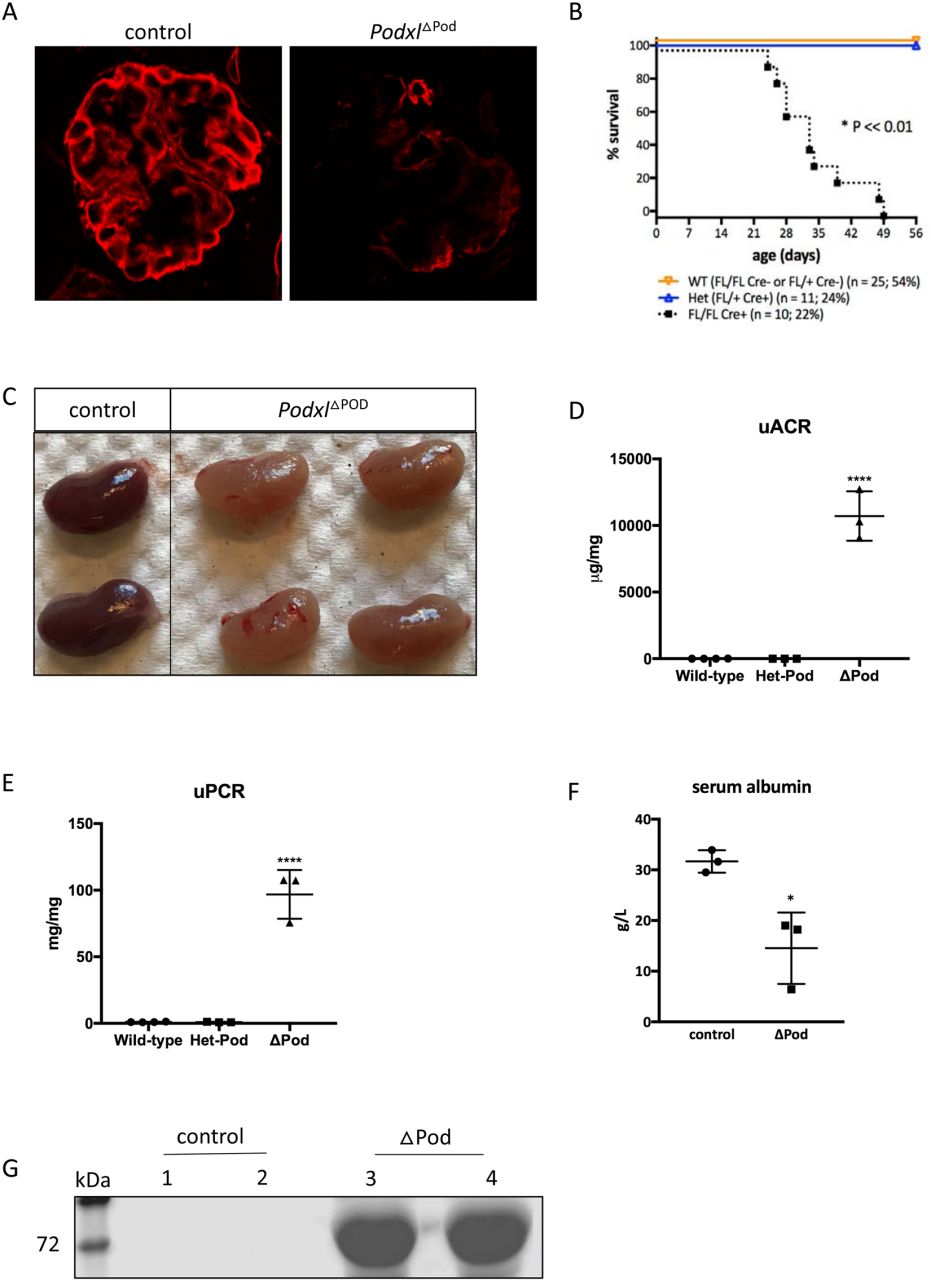
Loss of *Podxl* from podocytes leads to severe proteinuria and kidney failure at ~6–7 weeks after birth. (**A**) Anti-mouse Podxl immunostaining of glomerular sections from *Podxl*^△Pod^ and control mice. (**B**) Kaplan-Meier survival and Mendelian inheritance distribution from 8 litters with breeding scheme: *Podxl*^f/f^*Pod*-Cre^+/−^ X *Podxl*^f/+^*Pod*-Cre^−/−^. Expected distributions: *Podxl*^f/f^*Pod*-Cre^−/−^ OR *Podxl*^f/+^*Pod*-Cre^−/−^ (25% + 25% = 50%) ~ wild-type; *Podxl*^f/+^*Pod*-Cre^+/−^ (25%; *Podxl*^Het-Pod^); *Podxl*^f/f^*Pod*-Cre^+/−^ (25%; *Podxl*^△Pod^). Actual distributions: *Podxl*^f/f^ (n = 25; 54%), *Podxl*^Het-Pod^ (n = 11; 24%) and *Podxl*^△Pod^ (n = 10; 22%) groups. (**C**) Images of whole, unperfused kidneys from control and *Podxl*^∆Pod^ mice. (**D**) Urine albumin-creatinine ratio from 3-week-old wild-type (n = 4), *Podxl*^Het-Pod^ (n = 3) and *Podxl*^∆Pod^ (n = 3) littermates. ****p < 0.0001. (**E**) Urinary total protein-creatinine ratio from 3-week-old wild-type (n = 4), *Podxl*^Het-Pod^ (n = 3) and *Podxl*^∆Pod^ (n = 3) littermates. ****p < 0.0001. (**F**) Serum albumin concentration from 3-week-old controls (pooled wild-type and *Podxl*^Het-Pod^) (n = 3) and *Podxl*^∆Pod^ (n = 3) littermates. *p < 0.05. (**G**) Undiluted urine SDS-PAGE from wild-type (n = 2) and *Podxl*^△Pod^ (n = 2) mice. Bands at ~65–75 kDa correspond to albumin.

To evaluate the role of Podxl in differentiated podocytes, we generated a mouse strain carrying a floxed allele of the gene, enabling deletion via Cre recombinase (*Podxl*^f/f^)^1^. This *Podxl*^f/f^ strain was crossed with mice expressing Cre under the control of the podocyte-specific podocin (*Nphs2*) promoter, which initiates expression at the CLS of nephrogenesis (Pod-Cre)^21^. The resulting progeny (*Podxl*^ΔPod^ mice) bear a selective deletion of *Podxl* in morphologically mature podocytes permitting an opportunity to evaluate Podxl function after morphogenesis is complete. Glomeruli from 3-week-old *Podxl*^ΔPod^ mice exhibited a significant reduction in Podxl immunoreactivity (Fig. 1A). While *Podxl*^ΔPod^ mice survived gestation, by approximately 3 weeks of age, they appeared runted and exhibited a high rate of mortality thereafter, with 100% of mice reaching a humane endpoint by 7 weeks of age (Fig. 1B).

On visual inspection of age- and sex-matched litters containing *Podxl*^ΔPod^ and control mice at 4–6 weeks, we find that *Podxl*^ΔPod^ kidneys appeared pale and “flea-bitten” in comparison (Fig. 1C). Cortico-medullary sections from *Podxl*^ΔPod^ kidneys stained with H&E revealed deterioration of renal architecture (Suppl. Figure 1). Additionally, kidney injury molecule-1 (KIM-1), a diagnostic marker of kidney tubule damage^22^, was markedly increased in urine samples from *Podxl*^ΔPod^ mice (Suppl. Figure 2). This coincided with increased deposition of collagen I in *Podxl*^ΔPod^ kidneys (Suppl. Figure 3). Urine and serum analyses of *Podxl*^ΔPod^ mice revealed an increase in proteinuria (Fig. 1D–G), and a corresponding decrease in serum albumin (Fig. 1F). Taken together, these data indicate that podocyte-specific deletion of podocalyxin results in a congenital nephrotic syndrome that rapidly progresses to ESRD in adolescent *Podxl*^ΔPod^ mice.

### Renal pathology of *Podxl*^ΔPod^ mice closely resembles human FSGS

**Figure 2:**
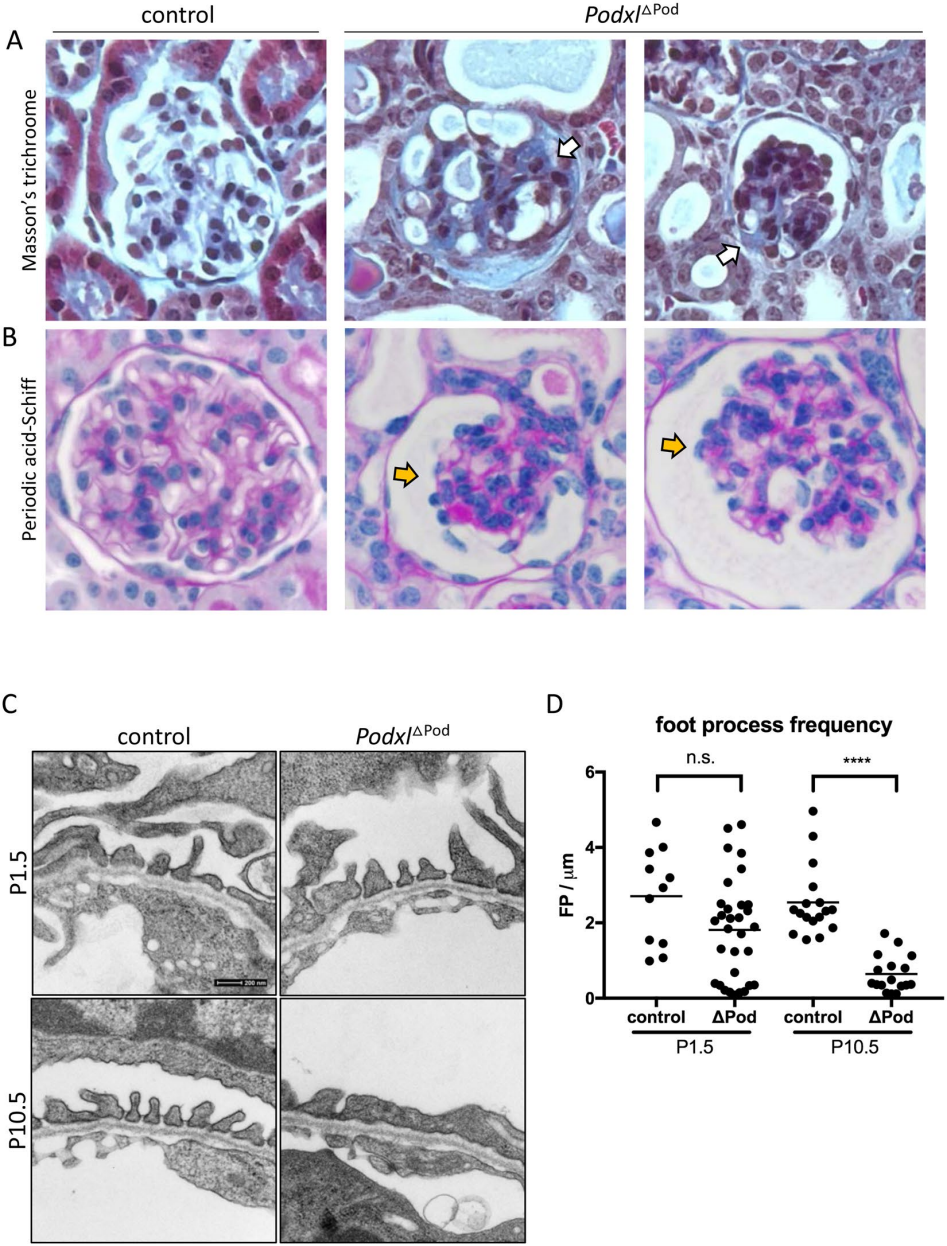
*Podxl*^△Pod^ mice present with progressive focal segmental glomerulosclerosis. (**A**) Masson’s trichrome stained kidney sections from *Podxl*^△Pod^ (n = 10) and control (n = 5) mice. (**B**) Periodic acid-Schiff stained kidney sections from *Podxl*^△Pod^ (n = 10) and control (n = 6) mice. (**C**) Transmission electron micrographs of podocyte foot processes in *Podxl*^△Pod^ and control mice at days 1.5 and 10.5 post-partum. (**D**) Quantification of foot process frequency (defined as continuous membrane events separated by a slit diaphragm per unit length of basement membrane) in podocytes from *Podxl*^△Pod^ and control mice. Number of TEMs quantified: control, P1.5 (n = 11), *Podxl*^△Pod^ P1.5 (n = 31), control, P10.5 (n = 17), *Podxl*^△Pod^ P10.5 (n = 17), Horizontal bars represent the mean. ****p < 0.0001.

We next conducted a detailed histopathological evaluation of glomerular lesions in *Podxl*^ΔPod^ mice and control littermates by Masson’s trichrome and periodic acid-Schiff (PAS) stains (Fig. 2A,B). *Podxl*^ΔPod^ mice showed histopathologic lesions of varying severities along the disease continuum at 3–5 weeks of age. We consistently observed several major phenotypes: segmental glomerulosclerosis was most common (Fig. 2A, white arrows) with lesions that were accompanied by collapsed capillary tufts (Fig. 2B, yellow arrows), and adhesions to the Bowman’s capsule. Furthermore, several mutant kidneys exhibited focal parietal epithelial cell (PEC) hyperplasia, and some glomeruli underwent complete or partial collapse. These findings coincided with a reduction in glomerular p57^+^ cells (Suppl. Figure 4), indicating a loss of differentiated podocytes^23^ in *Podxl*^ΔPod^ mice. In aggregate, these histological manifestations are consistent with the clinical presentation of human FSGS^24^.

### Podocytes in *Podxl*^ΔPod^ mice develop functional filtration slits but undergo effacement in the first weeks of life

Previously, we showed that mice bearing a germline deletion of *Podxl* die during the first 24 hours of postnatal life due to a defect in podocyte morphogenesis that leads to anuria and hypertension^9^. Specifically, differentiating podocytes failed to disassemble the lateral adhesion complexes between the adjoining nascent podocytes at the S-shaped body (SSB) phase of nephrogenesis. As a result, foot processes and slit diaphragms failed to form, resulting in a complete absence of urine production. In stark contrast, 100% of *Podxl*^ΔPod^ mice described here were fully capable of producing urine at birth (n = 6). At the ultrastructural level, transmission electron microscopy (TEM) revealed morphologically mature foot processes and slit diaphragms *Podxl*^ΔPod^ mice at birth, but these exhibited progressive effacement over the subsequent 10 days of postnatal life (Fig. 2C,D).

### Loss of Podxl from mature podocytes results in mislocalization of the slit diaphragm protein podocin

**Figure 3:**
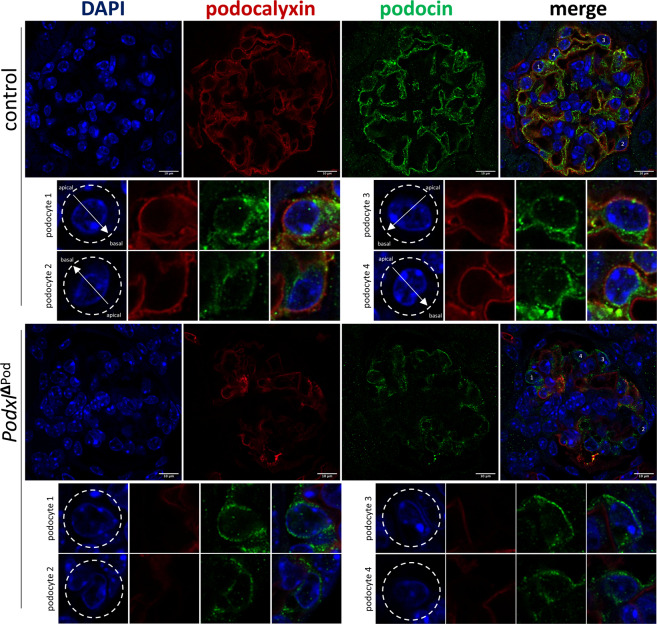
Podocin is mislocalized to the apical membrane domain in the absence of Podxl. Representative confocal micrographs from two experiments. Kidney sections (5 µm) from *Podxl*^△Pod^ and control mice co-stained for podocalyxin and podocin and imaged using a Zeiss Airyscan confocal microscope. In the control micrographs, arrows denote apical-basal polarity in control podocytes. In the *Podxl*^△Pod^ micrographs, lack of arrows indicates a loss of cellular polarity.

Previous studies have shown that Podxl plays a key role in apical membrane polarization in epithelial cell lines^25,26^. For example, in breast epithelial cells, Podxl overexpression leads to apical domain expansion, underscoring its importance in regulating apical membrane size and identity^25^. We therefore hypothesized that loss of Podxl may lead to a disruption of podocyte apical/basal polarity and mislocalization of foot process and slit diaphragm associated proteins, which are normally excluded from apical membrane domains of the podocyte cell body. To test this, we co-stained kidney sections from *Podxl*^ΔPod^ mice and controls for Podxl and the slit diagram protein podocin, and assessed their subcellular localization. While control podocytes exhibited the expected apical expression of podocalyxin and basolateral enrichment of podocin (Fig. 3, control panels), in mutant podocytes where podocalyxin expression is ablated, podocin expression was both reduced, and mislocalized to apical domains of podocyte cell bodies (Fig. 3, *Podxl*^ΔPod^ panels). These data suggest that in addition to regulating the morphogenesis of podocytes during development, Podxl also plays a later role in restricting slit diaphragm protein localization to the appropriate basal and lateral membrane domains in differentiated podocytes.

### Verification that inactivation of the *Podxl* gene in *Podxl*^ΔPod^ mice occurs in mature podocytes

**Figure 4:**
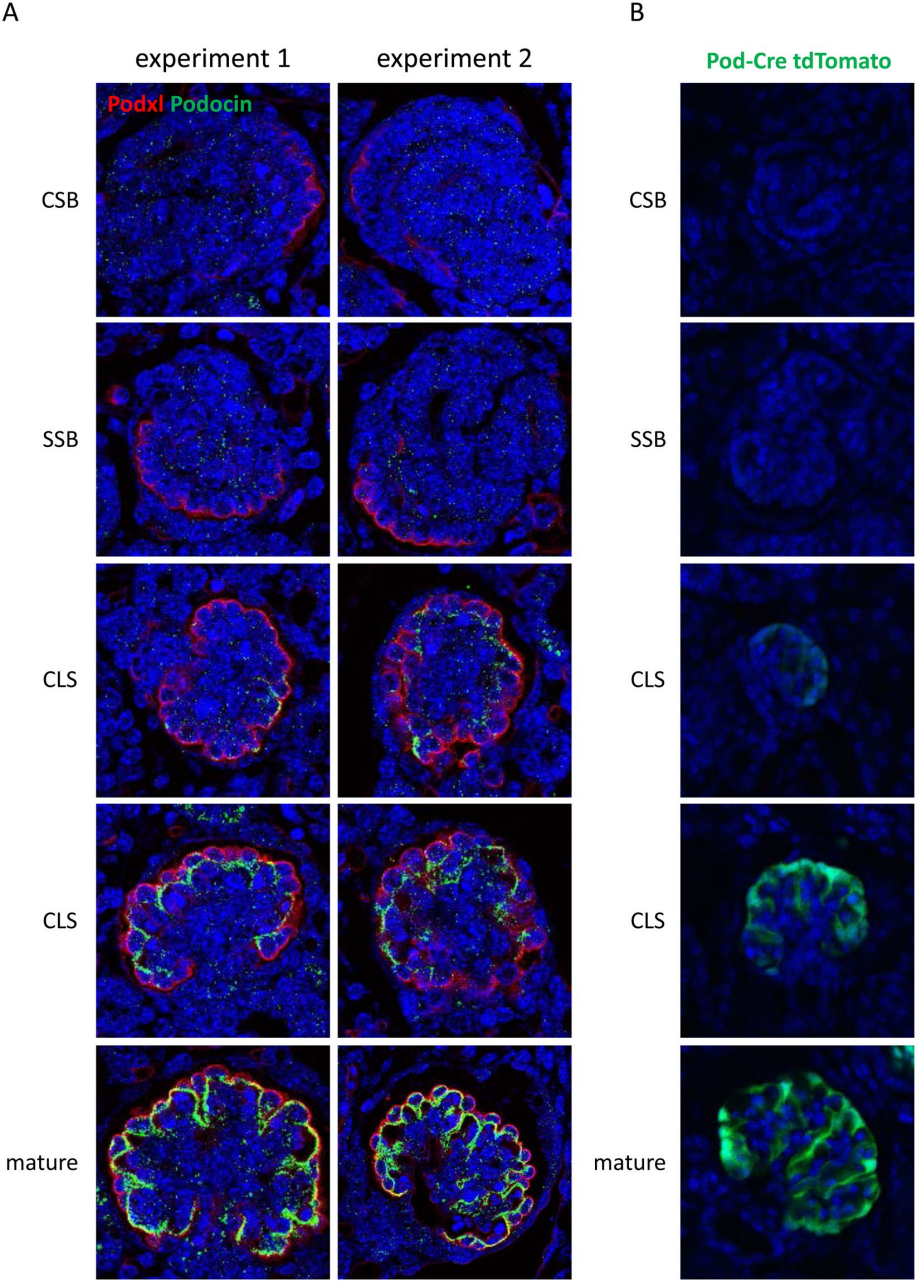
Podxl is expressed before podocin (and Cre in *Podxl*^△Pod^ mice) during nephrogenesis. (**A**) Representative confocal micrographs of Podxl (red) and podocin (green) expression in wild-type embryonic day 18.5 kidneys (n = 2 mice) from two separate experiments. Glomeruli are shown at the different stages of nephrogenesis. (**B**) Representative confocal micrographs showing native tomato fluorescence in post-natal day 1.5 kidneys from *Pod*-Cre^+/−^tdTomato^+/−^ mice (Cre-reporter strain). CSB, comma-shaped body; SSB, S-shaped body; CLS, capillary loop stage; Mature, fully matured glomerulus.

During fetal development, nephrogenesis occurs with the following temporal chronology: beginning as renal vesicles, developing nephrons mature through the “comma-shaped body” (CSB) phase, the “S-shaped body” (SSB), the “capillary loop stage” (CLS), until they finally form the adult glomerulus^27^. Podocytes first arise from SIX2^+^ progenitors at the SSB phase and facilitate vascular integration through VEGF-mediated signalling^27^. During this process, neighboring podocytes extend foot processes that interdigitate and become linked via remarkably specialized, semi-porous junctional complexes called slit diaphragms to form the glomerular filtration barrier. Previous knockout studies have suggested Podxl expression regulates this process, since germline *Podxl* deletion results in a lack of foot process formation and retention of impermeable junctions linking neighboring podocytes^9^. We hypothesized that the proteinuria and delayed lethality observed in *Podxl*^ΔPod^ mice likely reflects the fact that, in this strain, *Podxl* deletion occurs after its initial burst of expression in nascent SSB podocytes and after the cells have completed morphogenesis. To validate this interpretation, we co-stained kidneys harvested from embryonic day 18.5 (E18.5) C57BL/6 mice for Podxl and podocin. Kidneys at this stage of development harbour glomeruli at various stages of maturation, enabling us to examine glomeruli along all stages of the CSB→ SSB → CLS continuum by their appearance on the DAPI fluorescence channel. We found that Podxl expression first became apparent at the SSB, whereas podocin expression occurred later, at the CLS, after Podxl had been expressed by podocytes (Fig. 4A). Furthermore, using a reporter strain (ROSA26-stop/flox-tdTomato) to validate the time of Cre expression, we found that tdTomato fluorescence was detectable starting at the late CLS (Fig. 4B). In combination with our TEM observations, these data support the conjecture that ablation of Podxl in *Podxl*^ΔPod^ mice occurs after podocytes have reached the CLS and formed their characteristic mature ultrastructure required for filtration. Furthermore, this ‘delayed’ proteinuric phenotype is likely compounded by the finding that nephrogenesis ceases at day 6 after birth in mice^28^.

### *Podxl*^+/−^ mice have normal kidney architecture and function at steady state

**Figure 5:**
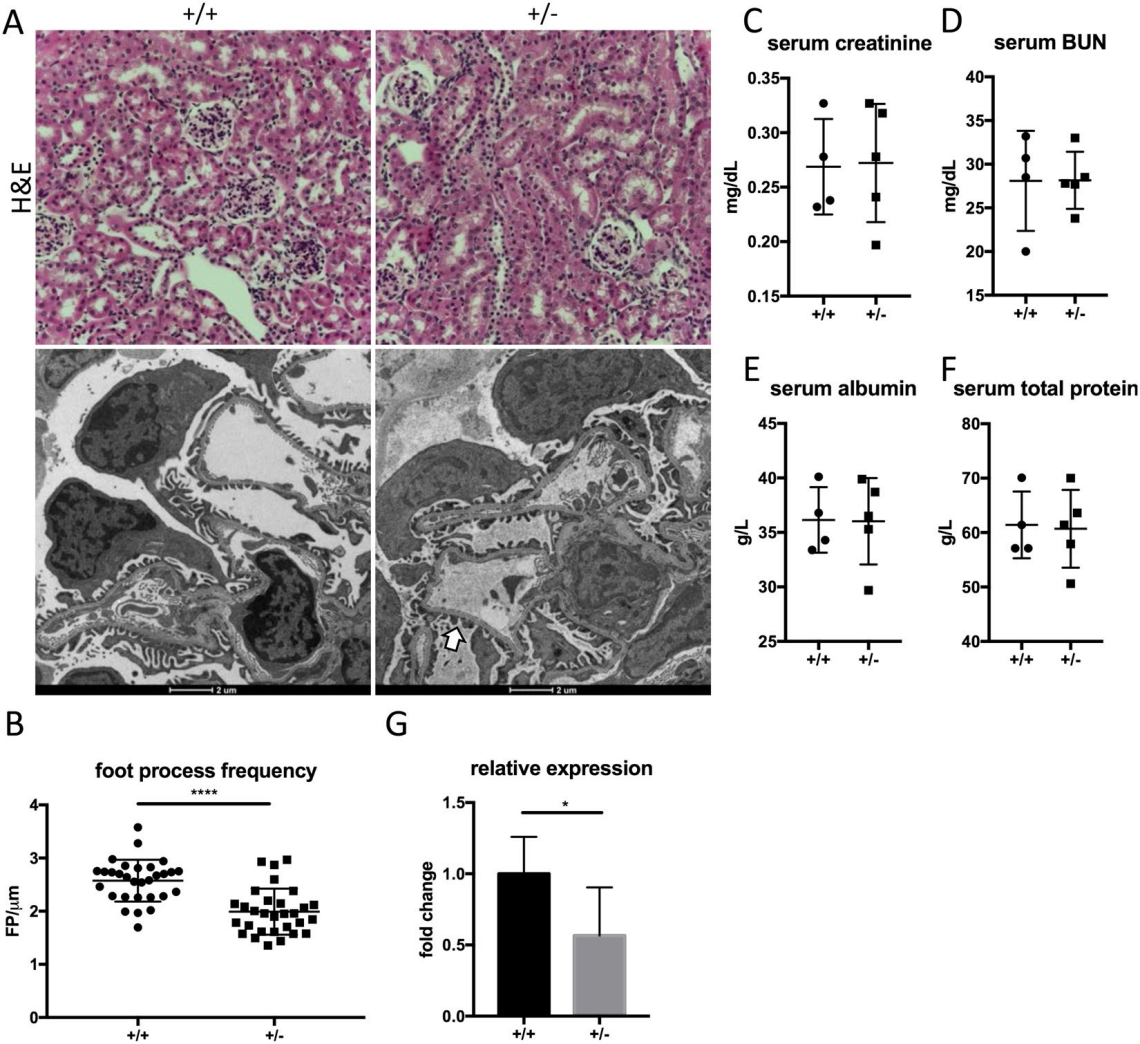
*Podxl*^+/−^ mice have normal kidney architecture and function. (**A**) H&E staining (upper panels) and transmission electron micrographs (lower panels) of cortical kidney sections from age- and sex-matched wild-type (n = 2) and *Podxl*^+/−^ (n = 2) mice. (**B**) Quantification of podocyte foot processes using transmission electron micrographs from age- and sex- matched wild-type (n = 2) and *Podxl*^+/-^ (n = 2) mice. ****p < 0.0001 (**C**) Serum creatinine quantification from age- and sex-matched wild-type (n = 2) and *Podxl*^+/-^ heterozygous (n = 2) mice. (**D**) Serum BUN quantification from age and sex matched wild-type (n = 2) and *Podxl*^+/-^ (n = 2) mice. (**E**) Serum albumin quantification from age- and sex-matched wild-type (n = 2) and *Podxl*^+/-^ (n = 2) mice. (**F**) Urine albumin:creatinine ratio from age- and sex-matched wild-type (n = 2) and *Podxl*^+/-^ (n = 2) mice. (**G**) Quantification of *Podxl* transcripts using whole-kidney RNA from wild-type (n = 4) and *Podxl*^+/-^ (n = 5) mice. Values are expressed as fold-change relative to the wild-type message. *p < 0.05 For all figure panels, 8-week old male and female littermates were examined.

Multiple human studies have implicated heterozygous nonsense or missense podocalyxin mutations in the pathogenesis of familial proteinuric kidney disease (18, 19 and reviewed in 20). We therefore sought to carefully characterize the renal architecture and glomerular filtration function of *Podxl*^+/−^ mice. Histological analysis of *Podxl*^+/−^ kidney sections showed no obvious difference in glomerular and tubulointerstitial architecture relative to wild-type controls (Fig. 5A, upper panels), and these mice have a normal lifespan. Examination of TEMs shows a subtle ‘ballooning’ of podocyte foot processes in *Podxl*^+/−^ mice (Fig. 5A, lower panels, white arrow) and quantification of foot process frequency revealed a slight reduction in filtration slits in *Podxl*^+/−^ mice relative to wild-type littermates (Fig. 5B). Next, we analyzed glomerular filtration function by measuring relevant serum biomarkers. There was no difference in serum creatinine, blood urea nitrogen (BUN), albumin and total protein between *Podxl*^+/−^ and wild-type mice (Fig. 5C–F). Finally, we analyzed whole-kidney RNA by quantitative real-time PCR, which showed a reduction in podocalyxin transcripts in *Podxl*^+/−^ mice relative to wild-type controls (Fig. 5G). In summary, these findings establish that adult *Podxl*^+/−^ mice have physiologically normal kidney function at steady state, but harbour subtle deviations in transcription and podocyte ultrastructure.

### *Podxl*^+/−^ mice are haploinsufficient with respect to chemically-induced nephrosis

**Figure 6:**
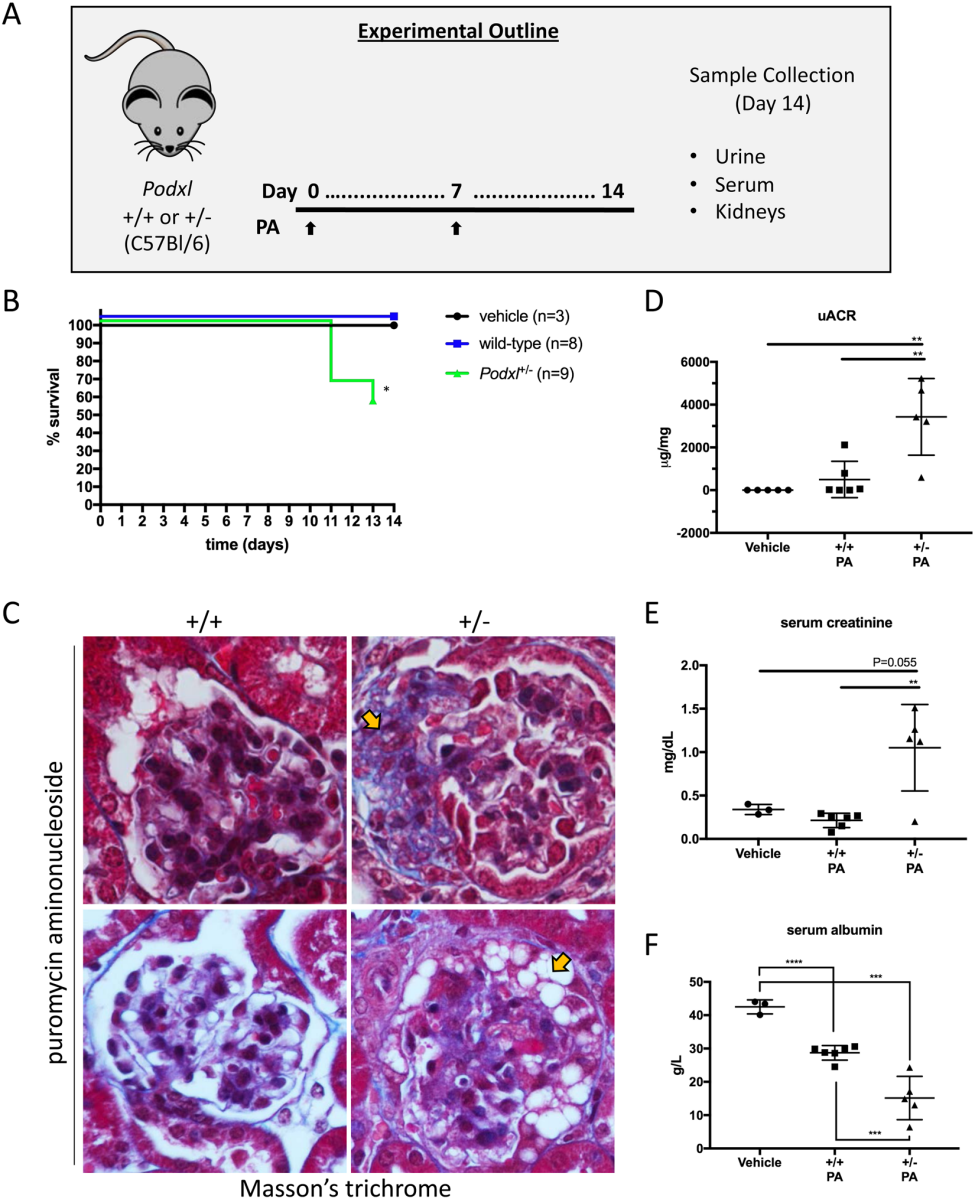
*Podxl*^+/−^ mice are haploinsufficient with respect to puromycin aminonucleoside nephrosis. (**A**) Schematic of experimental nephropathy in *Podxl*^+/−^ mice. Adult mice were treated with 2 intraperitoneal injections of puromycin aminonucleoside (450 mg/kg) on days 0 and 7. Sample collection occurred on day 14 (experimental endpoint). (**B**) Kaplan-Meyer survival analysis of wild-type and *Podxl*^+/−^ mice treated with 2 doses (Day 0, 7) of 450 mg/kg puromycin aminonucleoside (PA). Logrank test *p < 0.05. (**C**) Masson’s trichrome stains of cortical kidney sections from wild-type and *Podxl*^+/−^ mice treated with puromycin aminonucleoside. Yellow arrows on the upper and lower panels denotes segmental sclerosis and collapsing features, respectively. (**D**) Urine albumin:creatinine ratio from wild-type (n = 6) and *Podxl*^+/−^ (n = 5) mice treated with puromycin aminonucleoside and vehicle controls (n = 5). **p < 0.01 (**E**) Serum creatinine levels from wild-type (n = 6) and *Podxl*^+/−^ (n = 5) mice treated with puromycin aminonucleoside and vehicle controls (n = 3). **p < 0.01. (**F**) Serum albumin concentration from wild-type (n = 6) and *Podxl*^+/−^ (n = 5) mice treated with puromycin aminonucleoside and vehicle controls (n =3). ***p < 0.001; ****p < 0.0001.

**Figure 7:**
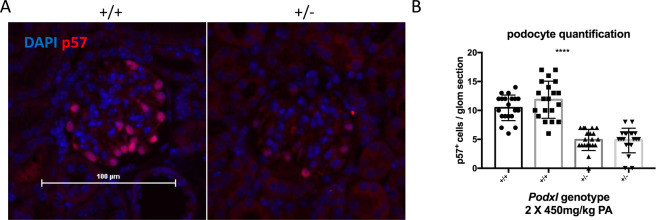
Mice treated with puromycin aminonucleoside undergo podocyte loss. (**A**) p57 immunofluorescence staining of 5 µm cortical kidney sections from wild-type (n = 2) and *Podxl*^+/−^ mice treated with puromycin aminonucleoside (n = 2). (**B**) Quantification of p57^+^ events per glomerular section. Multiple comparisons were performed between each wild-type and heterozygous sample, ****p < 0.0001.

Because heterozygous null mutations in humans have been linked to adult onset FSGS but *Podxl*^+/−^ mice are phenotypically normal, we hypothesized that podocalyxin hemizygosity alone may not be sufficient to cause renal disease. Rather, this mutation may render individuals and mice more susceptible to disease in conditions of stress or secondary environmental insults. To test this hypothesis, wild-type and *Podxl*^+/-^ mice were injected twice intraperitoneally with the nephrotoxin puromycin aminonucleoside (PA), on days 0 and 7, and monitored over a 14-day time course (Fig. 6A). Although all 8 wild-type mice treated with PA survived the time course as expected^29^, 5 out of 9 *Podxl*^+/−^ mice reached their humane endpoint during the course of the experiment (Fig. 6B). Histopathologic analyses of glomeruli from *Podxl*^+/−^ mice showed segmental lesions by Masson’s trichrome (Fig. 6C, upper panel, yellow arrow) and collapsing features (Fig. 6C, lower panel, yellow arrow), whereas the glomeruli of wild-type mice showed only slight hypercellularity (Fig. 6C, upper panel). Urine and serum analyses revealed severe proteinuria, elevated serum creatinine and a reduction in serum albumin in *Podxl*^+/−^ mice (Fig. 6D–F). Quantification of p57^+^ cells revealed a loss of differentiated podocytes in glomeruli of PA-treated *Podxl*^+/−^ mice relative to wild-type controls (Fig. 7A,B).

Next a blinded renal pathologist applied the Columbia classification to histological sections from this experiment and correlated the renal pathology of each specimen with its corresponding urine ACR value (Table 1). Overall, we found that PA-treated *Podxl*^+/−^ mice reliably develop collapsing glomerular pathologies with severe diffuse tubular injury. In contrast, PA-treated wild-type mice exhibited grossly unremarkable features, although rare not otherwise specified (NOS) and collapsing lesions were noted in 2 of 7 mice. Taken together, these findings support the conclusion that although they are normal at steady state, *Podxl*^+/−^ mice are highly susceptible to environmental insults and, in response to PA, develop severe proteinuria and a nephrotic syndrome similar to collapsing FSGS.

**Table 1:** Columbia classification of wild-type and *Podxl*^+/−^ mice challenged with puromycin aminonucleoside and their corresponding urine albumin:creatinine ratios.

n	Genotype	Tx Group	Variant	Pathology Phenotype	uACR (µg/mg)
1	+/+	vehicle	normal	Unremarkable.	1.53
2	+/+	vehicle	normal	Unremarkable.	2.02
3	+/+	vehicle	normal	Unremarkable.	1.93
4	+/+	PAN	normal	Unremarkable.	1.56
5	+/+	PAN	collapsing	Minimal ATI, FSGS, NOS and some with collapsing features.	2120
6	+/+	PAN	collapsing	FSGS, NOS and some with collapsing features but few FSGS lesions. Some tubular resorption droplets, small focus of interstitial inflammation.	790
7	+/+	PAN	NOS	FSGS, NOS with podocyte hypertrophy but no shrunken tufts. Acute tubular injury.	n/a
8	+/+	PAN	unremarkable	Rare podocyte resorption droplets. Otherwise unremarkable.	59.5
9	+/+	PAN	NOS	Rare FSGS in gloms with tuft shrinkage and protein in Bowman’s space. Frequent tubular and podocyte resorption droplets, Mild ATI.	n/a
10	+/+	PAN	unremarkable	FSGS, NOS lesion. Rare podocyte resorption droplets. Otherwise unremarkable.	n/a
11	+/+	PAN	unremarkable	Rare pocodyte resorption droplets. Otherwise unremarkable.	26.5
12	+/−	PAN	collapsing	FSGS, NOS with segmental sclerosis made up of hylaine and matrix material. Some with collapsing features. Some with podocyte/visceral epithelial cell hyperplasia with vacuoliation and podoctye resorption droplets. Some with shrinking of glomerular tufts and filling of Bowman’s space with proteinaceous material. Diffuse actue tubular injury	n/a
13	+/−	PAN	collapsing	Severe ATI. Shrunken tufts with proteinaceous material in Bowman’s space. FSGS, NOS with podocyte hypertrophy and vacuolization some with collapsing like lesions. Some tubular and podocyte resorption droplets.	3214
14	+/−	PAN	NOS	FSGS, NOS lesion. Rare podocyte resorption droplets. Otherwise unremarkable.	3422
15	+/−	PAN	collapsing	Severe ATI. Shrunken tufts with proteinaceous material in Bowman’s space. FSGS, NOS with podocyte hypertrophy and vacuolization some and collapsing like lesions. Some tubular and podocyte resorption droplets.	n/a
16	+/−	PAN	collapsing	Severe ATI. Shrunken tufts with proteinaceous material in Bowman’s space. FSGS, NOS with podocyte hypertrophy and vacuolization some and collapsing like lesions. Some tubular and podocyte resorption droplets.	5231
17	+/−	PAN	collapsing	Mild ATI. Some FSGS, NOS and collapsing, some glomeruli with shrinkage. Tubular and podocyte resorption droplets.	597.4
18	+/−	PAN	collapsing	Severe ATI. Shrunken tufts with proteinaceous material in Bowman’s space. FSGS, NOS with podocyte hypertrophy and vacuolization some and collapsing like lesions. Some tubular and podocyte resorption droplets.	4678

ATI, acute tubular injury; FSGS, focal segmental glomerulosclerosis; NOS, not otherwise specified; IF, interstitial fibrosis; TA, tubular atrophy.

For all cases: No IF/TA. All vessels normal. No/minimal global glomerular sclerosis. ATI associated with numerous proteinaceous casts with similarities to microcystic changes in severe cases.

n/a, urine not collected either due to severe disease (in case of *Podxl*^+/-^ mice) or exceeding humane urine collection time window (in case of wild-type mice).

## Discussion

**Figure 8:**
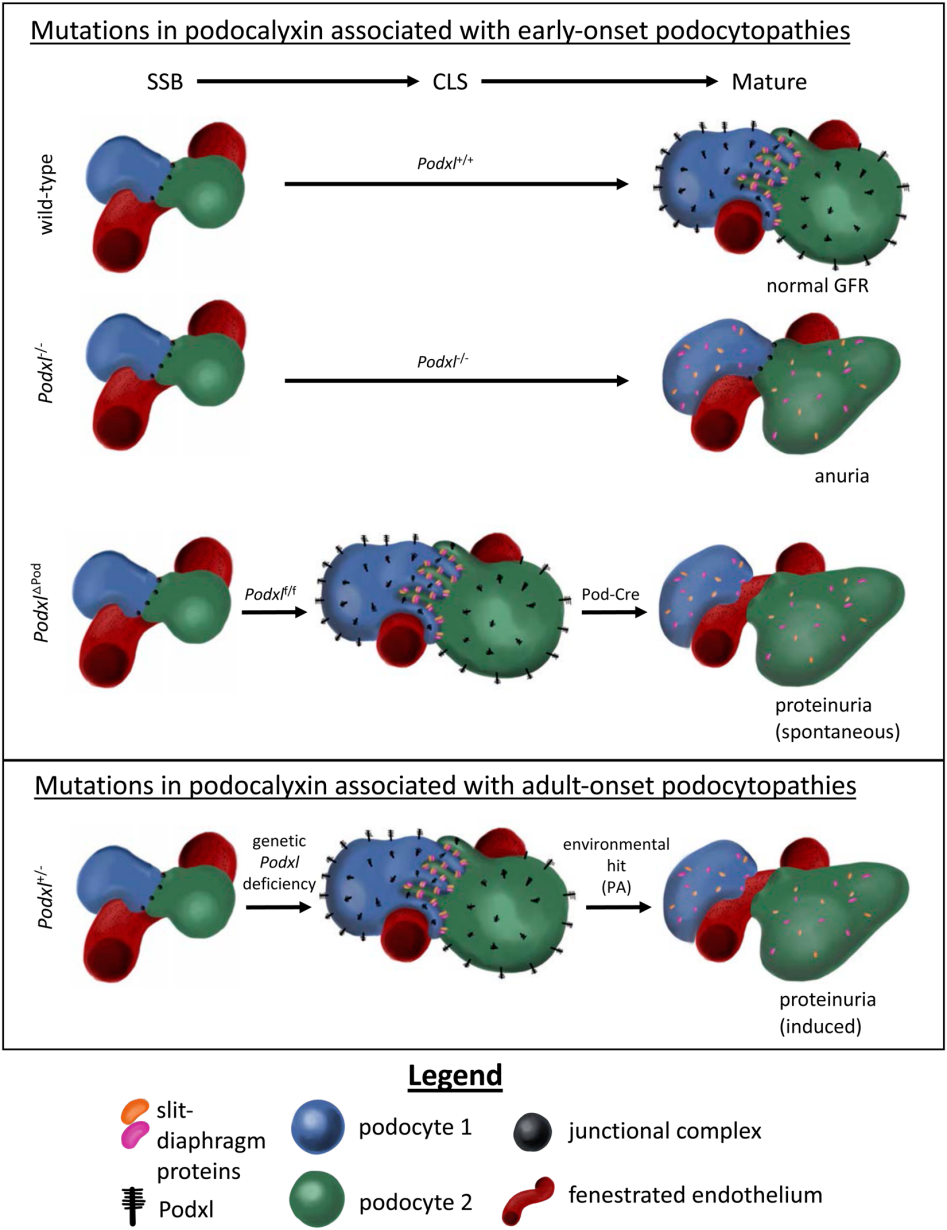
The timing of Podxl deletion in podocytes affects the observed glomerular filtration phenotype. In wild-type mice, expression of Podxl at the SSB resolves the lateral junctions adjoining neighboring podocytes, enabling the expression and correct localization of slit diaphragm proteins. In *Podxl*^−/−^ mice, failure to express Podxl renders podocytes incapable of resolving their junctional complexes. As a result, slit diaphragm proteins are expressed but fail to localize selectively to the basal domain, leading to an absence of filtration slit surface area. These mice therefore lack the ability to produce any urine. In *Podxl*^△Pod^ mice, Podxl is expressed at the SSB, and thus podocytes are able to resolve their junctional complexes, allowing for the initial formation of slit diaphragms at the basal membrane domain. When Podxl is deleted by Cre at the CLS, slit diaphragm proteins mislocalize apically. Due to the high hydrostatic pressure generated by the perfusion of glomerular capillaries and the absence of adherens junctions on podocytes, slit diaphragms do not wall off. Instead, podocyte effacement occurs, leading to proteinuria. In *Podxl*^+/−^ mice, the reduction in *Podxl* transcripts does not affect podocyte differentiation and they are able to complete morphogenesis similar to wild type podocytes, resulting in normal glomerular filtration at steady state. Following a second environmental hit, mutant podocytes are haploinsufficient and  undergo effacement. As a result, they develop FSGS and proteinuria.

In this study, we addressed the role of podocalyxin in mature podocytes. Deletion of the gene at the CLS of nephrogenesis using conditionally-expressed Cre recombinase led to death between 3 to 7 weeks of age from a severe congenital nephrotic syndrome characterized by FSGS and proteinuria. This phenotype is strikingly different from the anuria and perinatal lethality observed previously in the germline *Podxl*^−/−^ mice^9^. A key difference between these two strains is the developmental time point at which the *Podxl* gene is inactivated in podocytes. In the previous report^9^, germline *Podxl* deletion revealed an indispensable role in regulating the dissolution of junctional complexes adjoining immature podocytes at the SSB phase of nephrogenesis, a process that is required for the formation of filtration slits (shown schematically in Fig. 8). While this revealed a critical function for Podxl in podocyte morphogenesis, it failed to test whether Podxl serves an additional role in the maintenance of mature, differentiated podocytes. Here we show that, indeed, Podxl is required by mature podocytes to (1) maintain foot process architecture (both foot processes and slit diaphragms are lost in the mutants), (2) appropriately target the localization of foot process and slit diaphragm proteins to the correct membrane domains (podocin is mislocalized to apical domains in the mutants), and (3) maintain the structural integrity required for ultrafiltration (as evidenced by the severe proteinuria in mutant animals). As shown schematically in Fig. 8, normally, expression of Podxl at the SSB leads to a remodelling and partial dissolution of junctional complexes between neighboring podocytes, and thereby enables the formation of foot processes and slit diaphragms. Germline deletion of the *Podxl* gene ablates this key step in podocyte morphogenesis, leading to subsequent anuria, hypertension and perinatal death. In contrast, delayed Cre-mediated deletion of Podxl in *Podxl*^ΔPod^ mice enables podocytes to complete this first key step in morphogenesis as they are endowed with Podxl expression at the SSB, and instead leads to loss of Podxl from structurally mature podocytes. Thus, it permits the evaluation of a secondary role in maintenance of the architecture of these cells. We surmise that because podocytes lack expression of junctional proteins at this stage, loss of Podxl does not result in walling off of the urinary space and anuria. Instead, the powerful hemodynamic forces that perfuse the fenestrated endothelium contribute to podocyte effacement, slit diaphragm protein mislocalization and loss of this ultrastructure leads to proteinuria and progressive FSGS.

Previously, Barua *et al*. used whole exome sequencing to identify a non-synonymous mutation in PODXL’s transmembrane domain that co-segregated with familial, autosomal dominant FSGS^18^. Afflicted individuals were heterozygous for a variant that resulted in a missense mutation, converting a highly conserved non-polar leucine residue to a charged arginine (p.L442R) in PODXL’s transmembrane domain. This arginine substitution facilitated homodimerization of PODXL proteins at the cell membrane when expressed in MDCK cells^18^. Because the *Podxl*^ΔPod^ mice described here largely phenocopy this syndrome, we would argue that this human mutant retained sufficient function of PODXL (from the mutant or non-mutant allele) to permit podocyte morphogenesis (i.e., dissolution of cell-cell junctions and formation of foot processes and slit diaphragms), but not the subsequent functional requirements for maintenance of foot process architecture and slit diaphragm protein localization over the life course. Thus, *PODXL* variants in the affected individuals likely produced the extracellular repulsive charge necessary to remodel the junctional complexes characteristic of early SSB podocytes, however, aberrant dimerization of PODXL at the membrane may disrupt key protein binding interactions necessary for the maintenance of functional podocyte morphology.

Recently, heterozygous loss-of-function mutations in human PODXL were linked to late onset familial renal insufficiency in two distinct pedigrees from disparate ethnic backgrounds^18,19^. Our investigation of *Podxl*^+/−^ mice at steady state indicates that, although their kidneys are histologically and physiologically normal, they harbour subtle deviations in podocyte biology (reduced foot process frequency and podocalyxin expression) that render them susceptible to a “second hit”. Indeed, treatment of *Podxl*^+/−^ mice with PA, a chemical to which wild-type C57Bl/6 mice are intrinsically resistant^29^, induces a nephrotic syndrome strikingly similar to collapsing FSGS with severe proteinuria. This supports the study by Lin and colleagues described above, in which a heterozygous mutation in *PODXL* was associated with a late-onset form of FSGS. These results are also reminiscent of previously reported genetic lesions which can lower the threshold for kidney disease, such as variants in the *APOL1* gene and HIV-associated nephropathy (HIVAN)^30^. PA-treated *Podxl*^+/−^ mice may prove to be a robust model for proteinuric kidney disease that can be exploited for the identification of novel early biomarkers of FSGS or for pre-clinical therapeutic screening. They also offer the opportunity to evaluate steroid sensitivity, dietary modifications and immune modulation, all of which have been shown to be modifiers of human disease progression. Additionally, because of its penetrance in a C57Bl/6 congenic background, *Podxl*^+/−^ mice offer an attractive tool for the identification of modifier genes due to the extensive number of mutants available in this genetic background.

With respect to a wider role for podocalyxin in kidney disease, it is noteworthy that soluble urinary PODXL has been consistently documented in patients with podocytopathies in a wide range of renal disorders. Indeed, this has been used as a biomarker of podocyte injury in lupus nephritis^31^, diabetic nephropathy^32,33^, obesity^34^, and hypertension^35^. Based on the data presented here, we would argue that urinary Podxl may not be merely a biomarker^20^. Perhaps loss of Podxl in the urine heralds the loss of a key molecule required for podocyte function and the progression to a maladaptive state of membrane remodelling, loss of podocyte adhesion and function. Conversely, monitoring of candidate therapeutics that can mitigate the shedding of urinary PODXL and retain its expression on the surface of podocytes, is likely an effective strategy for gauging their efficaciousness in repairing podocyte architecture and restoring glomerular filtration function.

## Methods

### Generation of *Podxl*^ΔPOD^ mice

All mice in this study are C57Bl/6 congenic (backcrossed > 8 generations using C57Bl/6 J). B6.Cg-Tg(NPHS2-Cre)295-Lbh/J (JAX# 008205), referred to in this manuscript as *Pod*-Cre mice, were purchased from the Jackson Laboratory (Bar Harbour, ME, USA) and crossed with a B6-congenic *Podxl*^+/−^ strain generated from our previously described *Podxl*^f/f^ strain^1^ using the female germline loxP deletion property of the B6.Cg-Tg(TekCre)12Flv/J strain (JAX#004128). Subsequent selective intercrossing eliminated the TekCre allele and generated a stable *Podxl*^+/−^ strain expressing two *Pod*-Cre alleles. Males from this strain were crossed to *Podxl*^f/f^ females to generate *Pod*-Cre expressing experimental mice and littermate controls. B6.Cg-*Gt(ROSA)26Sor*^*tm9(CAG-tdTomato)Hze*^/J (JAX# 007909, The Jackson Laboratory), designated tdTomato mice, were used to generate a *Pod*-Cre reporter strain. Male *Pod*-Cre^+/+^ (Cre homozygous) mice were crossed with female tdTomato (homozygous) reporter mice to generate experimental offspring. All mouse experiments were conducted humanely with approval of the University of British Columbia’s Animal Care Committee (Protocol #A18–0121 (breeding), A18–0123 (necropsy), and A18–0352 (PAN)) based on ethical guidelines provided by the Canadian Committee on Animal Care.

### Puromycin aminonucleoside nephropathy

C57Bl/6-cg wild-type and podocalyxin-heterozygous mice were treated with two 450 mg/kg doses of puromycin aminonucleoside (P7130, Sigma-Aldrich) by intraperitoneal injection. The first injection was performed at day 0 of experiments, and the second on day 14. Mice were monitored as per the animal care guidelines of the University of British Columbia, and sacrificed at the experimental endpoint (day 14) or upon reaching a humane endpoint.

### Kidney tissue harvest and fixation

Mice were sacrificed by CO_2_ asphyxiation. Excised kidneys were then washed with ice-cold PBS and imaged using a standard 16-megapixel camera. For immunohistochemistry studies, mice were sacrificed and immediately perfused with 20 ml of 2 mM EDTA in PBS and 20 ml of 4% paraformaldehyde (PFA) prepared in PBS. Kidneys were removed and immersed in 4% PFA overnight and transferred into 75% ethanol solution and stored at 4 °C until paraffin-embedding. For electron microscopy studies, mice were sacrificed, perfused with 10 ml of 2 mM EDTA in PBS and subsequently with 10 ml of EM fixative solution (1.5% PFA, 1.5% glutaraldehyde in 0.1 M sodium cacodylate buffer, pH 7.3). Kidneys were then excised, sliced into 3 pieces and placed in the same fixative at room temperature. After 2–4 h, the fixative was replaced with buffer (0.1 M sodium cacodylate at pH 7.3) and stored at 4 °C until further processing.

### Histochemical staining

Whole, paraffin-embedded kidneys were sectioned at 5 µm and prepared for histochemical analysis by the Biomedical Research Centre’s in-house histology facility or Wax-it Histology Services Inc. The kidney sections were then imaged using a bright-field microscope (Nikon Corporation, Japan).

### Immunofluorescence staining

All immunostaining was carried out on 5 µm sections of paraffin-embedded kidneys. Kidney sections were deparaffinized in xylenes, hydrated in a series of descending ethanol concentrations (100%, 95%, 70%) and then washed in water for >3 min. Antigen retrieval was performed by immersing the slides in sodium citrate buffer (pH 6) at >90 °C for 30 min, and cooled to room temperature. After antigen retrieval they were washed in 0.05% Tween 20 in PBS, blocked for 20 min and stained with primary antibody overnight at 4 °C in a dark chamber – anti-collagen I (2 µg/ml, Abcam, ab21286); anti-mouse Podxl (2 µg/ml, R&D Systems, Minneapolis, MN, USA); anti-p57 (1 µg/ml, Santa Cruz, sc-82980); anti-podocin (1 µg/ml, Abcam, ab50339). The primary antibody solution was then washed with 0.05% Tween-20 in PBS and the slides were stained with an Alexa Fluor-conjugated secondary antibody (a concentration of 4 µg/ml was used for all secondary antibodies used in this manuscript) for 60 min at room temperature. Lastly, slides were washed with 0.05% Tween-20 in PBS, incubated with DAPI reagent (600 nM, catalog # D3571, Thermo Fisher Scientific) for 5 min and mounted in ProLong Gold antifade reagent (P36934, Thermo Fisher Scientific Inc., Fife, WA, USA). For slides stained with Collagen I, imaging was done using an epifluorescence microscope using the TexasRed Channel (Nikon Corporation, Japan). For all other stains, imaging was performed using a confocal microscope (LSM900 Zeiss Airyscan Confocal Microscope, Germany).

### Transmission electron microscopy

Samples were washed twice for 10 min each with buffer, and then post-fixed for 1 h on ice in 0.1 M sodium cacodylate and 1% osmium tetroxide (Electron Microscopy Sciences, Hatfield PA, United States). The tissue was then washed 3×, 10 min each with ddH_2_O, stained for 1 h with uranyl acetate (Canemco-Marivac Inc., Lakefield, Quebec, Canada), washed another 3X with ddH_2_O, and then dehydrated using a series of ascending concentrations of ethanol solutions. The ethanol was replaced with 100% propylene oxide and then the samples were left overnight in 1:1 propyleneoxide and Embed-812 (EMS, Hatfield, PA). The following day, samples were embedded in 100% Embed-812 and then polymerized at 60 °C for 48 h. The blocks were sectioned and then the sections were stained both with uranyl acetate and lead citrate. Sections were imaged using a Tecnai G2 Spirit electron microscope (FEI North America NanoPort, Hillsboro, OR) operated at 120 kV. Since nephrogenesis ceases at day 6 post-partum, for experiments conducted on *Podxl*^ΔPOD^ mice, only mature glomeruli were selected for analysis and quantification. This was done based on their appearance, selecting for only those having mature capillary tufts.

### KIM-1 Immunoassay

Urinary KIM-1 from *Podxl*^ΔPOD^ and *Podxl*^HET-POD^ mice was measured using the Quantikine ELISA Mouse TIM-1/KIM-1/HAVCR Immunoassay (R&D Systems Inc., MN, USA; Catalog No. MKM100) as per the manufacturer’s instructions. Urine was collected using Labsand (Coastline Global Inc., CA, USA) by placing mice atop the cage wire of a sanitized cage filled with Labsand, and collecting the urine using a pipette after it had accumulated in the cage. Importantly, the cages were monitored constantly, and fecal matter, if present, was removed from the cage every 5 min to prevent urine contamination. After collection, urine was immediately stored at −20 °C. Prior to assaying, urine was centrifuged at 4 °C to remove debris from the samples.

### Urine SDS-PAGE

Urine was collected using Labsand (Coastline Global Inc., CA, USA) by placing mice atop the cage wire of an empty cage filled with Labsand and pipetting the urine after it has collected at the bottom of the cage. Importantly, the cages were monitored constantly, and fecal matter, if present, was removed from the cage every 5 min to prevent urine contamination. After collection, urine was immediately stored at −20 °C. Prior to assaying, urine was centrifuged to remove debris from the samples. Undiluted urine was run by protein gel electrophoresis using a 4% stacking gel (for 15 min at 80 V) and a 10% separating gel (for 1 h at 100 V).

### Urine and serum analyses

Urine was collected using the Labsand method (described above). Serum was prepared by first collecting blood by cardiac puncture and allowing it to clot on ice for ~2 h. Samples were then centrifuged at 6000 x*g* at 4 °C for 10 min, and the supernatant (serum) was extracted from the sedimented mixture by pipetting. Biomarker analysis was performed at The Centre for Phenogenomics (Toronto, ON).

### Image quantification and statistics

For foot process quantifications, a single foot process was defined as a continuous membrane event separated by a slit diaphragm per unit length of the glomerular basement membrane. Approximately 2 to 10 microns of basement membrane length were considered for each measurement. Foot processes were quantified using transmission electron micrographs and the ImageJ software platform. Statistical analysis was performed using a two-tailed t test for samples control and mutant cohorts for both P1.5 and P10.5 time points. For the quantification of p57^+^ cells, sections from control (n = 4) and mutant (n = 7) mice were stained in duplicates and imaged using an epifluorescence microscope (Nikon, Japan). The number of p57^+^ cells per glomerular section was quantified for each section. In total, 100 control glomeruli and 175 mutant glomeruli were analyzed. The data was then pooled and statistical analysis was performed using a two-tailed t test comparing control and mutant groups.

## Supplementary information

Supplementary Figure Legends

Supplementary Figures
