# Supplementary material for: Distinct Functional Requirements for Podocalyxin in Immature and Mature Podocytes Reveal Mechanisms of Human Kidney Disease
Source: Sci Rep. 2020 Jun 10;10:9419. doi: 10.1038/s41598-020-64907-3 (PMC7286918; doi:10.1038/s41598-020-64907-3)
Supplement: Supplementary file 1 — Supplementary Figure Legends [file 41598_2020_64907_MOESM1_ESM.docx]

Supplementary Figure 1: Severe deterioration of kidney architecture in *Podxl*^△Pod^ mice. Representative images of H&E-stained kidney sections from *Podxl*^△POD^ and control mice.

Supplementary Figure 2: Urinary KIM-1 is upregulated in *Podxl*^△Pod^ mice. Quantification of urinary KIM-1 in *Podxl*^△Pod^ (n=3) and control (n=2) mice. P=0.0024.

Supplementary Figure 3: Podxl^△Pod^ mice develop renal fibrosis. Immunofluorecence microscopy for collagen I showing periglomerular and interstitial collagen deposition.

Supplementary Figure 4: Podocyte loss in *Podxl*^ΔPod^ mice. Quantification of p57^+^ cells per glomerular section. Pooled data from control (n=4) and *Podxl*^ΔPOD^ (n=7) mice. Error bars represent the mean ± SD. P<0.0001.
