# Supplementary material for: Distinct Functional Requirements for Podocalyxin in Immature and Mature Podocytes Reveal Mechanisms of Human Kidney Disease
Source: Sci Rep. 2020 Jun 10;10:9419. doi: 10.1038/s41598-020-64907-3 (PMC7286918; doi:10.1038/s41598-020-64907-3)

# Supplementary Figure 1

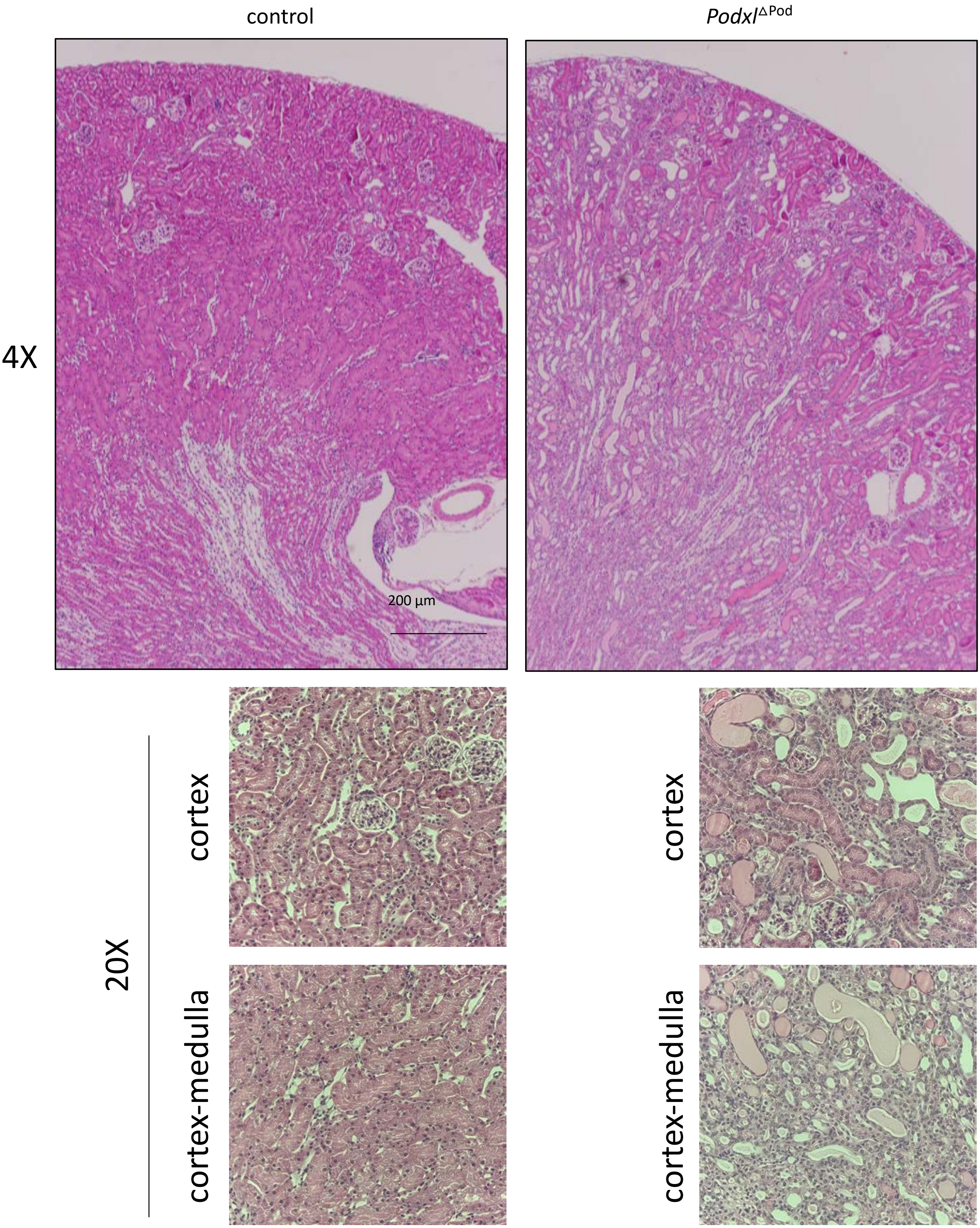

Supplementary Figure 2

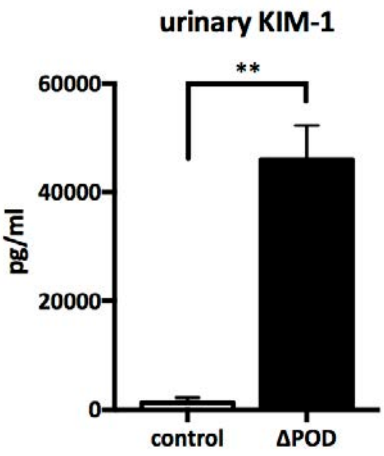

# Supplementary Figure 3

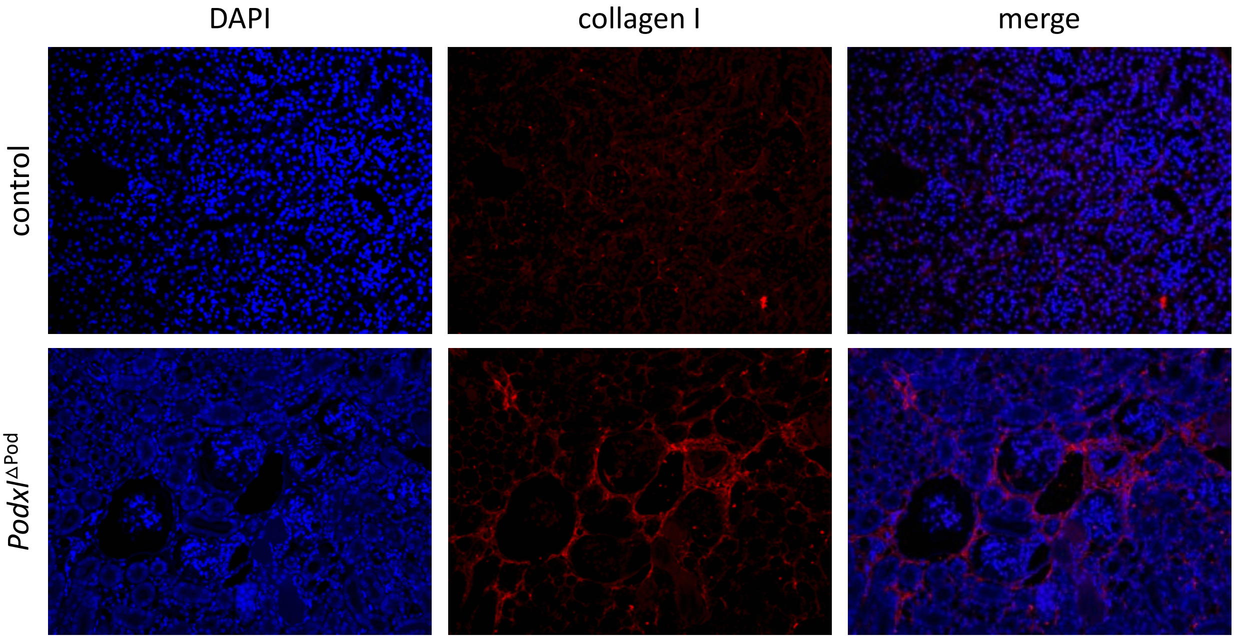

# Supplementary Figure 4

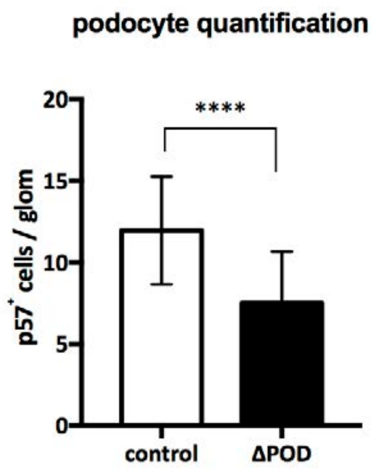

Supplement: Supplementary file 2 — Supplementary Figures [file 41598_2020_64907_MOESM2_ESM.pdf]
